# Insights into the Molecular Events That Regulate Heat-Induced Chilling Tolerance in Citrus Fruits

**DOI:** 10.3389/fpls.2017.01113

**Published:** 2017-06-26

**Authors:** María T. Lafuente, Beatriz Establés-Ortíz, Luis González-Candelas

**Affiliations:** Department of Biotechnology, Instituto de Agroquímica y Tecnología de Alimentos (CSIC)Valencia, Spain

**Keywords:** cold stress, fruit physiology, gene expression, heat-conditioning, oxidative stress, physiological disorder, transcriptome, WRKY

## Abstract

Low non-freezing temperature may cause chilling injury (CI), which is responsible for external quality deterioration in many chilling-sensitive horticultural crops. Exposure of chilling-sensitive citrus cultivars to non-lethal high-temperature conditioning may increase their chilling tolerance. Very little information is available about the molecular events involved in such tolerance. In this work, the molecular events associated with the low temperature tolerance induced by heating Fortune mandarin, which is very sensitive to chilling, for 3 days at 37°C prior to cold storage is presented. A transcriptomic analysis reveals that heat-conditioning has an important impact favoring the repression of genes in cold-stored fruit, and that long-term heat-induced chilling tolerance is an active process that requires activation of transcription factors involved in transcription initiation and of the WRKY family. The analysis also shows that chilling favors degradation processes, which affect lipids and proteins, and that the protective effect of the heat-conditioning treatment is more likely to be related to the repression of the genes involved in lipid degradation than to the modification of fatty acids unsaturation, which affects membrane permeability. Another major factor associated with the beneficial effect of the heat treatment on reducing CI is the regulation of stress-related proteins. Many of the genes that encoded such proteins are involved in secondary metabolism and in oxidative stress-related processes.

## Introduction

Storage at low non-freezing temperature is necessary to extend the postharvest life of horticultural crops because it delays senescence and reduces water loss and decay. Moreover, low-temperature treatments are required for pest control in quarantine treatments. Nevertheless, storage at temperatures below 12°C may cause injury in many chilling-sensitive crops of tropical and subtropical origin (Sevillano et al., 2009).

Fruits of many citrus cultivars are very prone to develop chilling injury (CI) (Mulas and Schirra, 2007). Susceptibility to CI vastly differs among species. The incidence of this physiological disorder in citrus fruit also depends on pre-harvest factors, including maturation stage and environmental conditions during fruit growth (Lafuente et al., 1997), and on field temperatures prior to cold storage (Gonzalez-Aguilar et al., 2000). This might partly explain variations in the seasonal development of CI among chilling-susceptible citrus fruits, such as grapefruits and mandarins harvested in different geographical regions (Purvis et al., 1979; Lafuente et al., 1997).

Citrus fruits are the highest value fruit crop in terms of international trade. Current annual worldwide citrus production is estimated at over 137 million tons (www.fao.org/faestat) and about two-third of citrus fruit production goes for fresh consumption. Important economic losses can occur in citrus fruit because of the manifestation of CI that affects external fruit quality due to the appearance of aesthetic defects, manifested as peel pitting (Sanchez-Ballesta et al., 2003) and superficial scald (Alférez et al., 2005) on the outer colored part of the peel (flavedo).

Considerable efforts have been made to develop strategies that reduce the incidence of CI in horticultural crops (Saltveit, 1991; Wang, 1993; Lurie, 1998; Sevillano et al., 2009). Understanding the mechanisms that underlie the beneficial effects of these strategies, and the influence of pre-harvest factors (Ferguson et al., 1999; Pedreschi and Lurie, 2015), would help to develop more feasible methods to extend the postharvest storage of citrus fruits. Pre-storage temperature conditioning is the most important means of increasing chilling tolerance in citrus fruits. Temperature-conditioning methods include using: (a) intermittent warming, i.e., periodic warming above the chilling temperature; (b) application of intermediate temperatures between growing and chilling temperatures (hardening); (c) non-lethal high temperatures (Saltveit, 1991; Wang, 1993; Lurie, 1998; Schirra and Cohen, 1999). High temperature conditioning is applied by using hot humid air (HA) (Martinez-Tellez and Lafuente, 1997; Ferguson et al., 2000) or hot water dip (HWD) treatments (Wild, 1993; Rodov et al., 1995; Schirra et al., 1997). Conditioning citrus fruits with HA for 3 days at about 37°C has been consistently found to be very effective in increasing chilling tolerance without inducing heat damage. This has been demonstrated for different citrus seasons and in fruits harvested at all maturity stages, despite the variable susceptibility of fruit throughout the season (Lafuente et al., 1997; Holland et al., 1999; Gonzalez-Aguilar et al., 2000). The excellent efficacy and reproducibility of the 3-day treatment at 37°C, named curing, has been shown with Fortune mandarins (hybrid of “Dancy” mandarin x “Clementine” mandarin) as a model of study because of its high susceptibility to chilling. Therefore, this cultivar and the availability of this HA treatment have provided a very valuable tool to study the physiological mechanisms that underlie long-term heat-induced chilling tolerance in citrus fruits, and also the influence of pre-harvest factors.

Physiological studies have provided very valuable information about the involvement of hormones, oxidative stress, lipids, carbohydrates, and phenolics metabolism in the susceptibility of citrus fruits to chilling and also in the heat-induced chilling tolerance (Lafuente et al., 2005). Moreover, physiological studies have demonstrated that pre-harvest conditions have a strong effect on the heat-induced responses in cold-stored citrus fruits. Thus, for a similar CI index, the more mature the fruit, the greater the cold-induced shift in the activity of the enzyme PAL, at the entry point of phenylpropanoids metabolism (Lafuente et al., 2003). Maturity or pre-harvest environmental conditions may also influence other chilling- or heat-induced responses as changes that occur in abscisic acid (ABA) (Lafuente et al., 1997), polyamines (Gonzalez-Aguilar et al., 1998, 2000), or in carbohydrates metabolism (Holland et al., 2002). These results envisage that the CI problem is not a simple one and indicate that heat-induced chilling tolerance in citrus fruit appears to be an active process that requires the activation of complex mechanisms, which can vary with fruit maturity stage or other pre-harvest factors.

Studies on molecular events related to citrus fruit tolerance to chilling began at the beginning of the twenty-first century. Different stress-related genes, induced by cold stress or by temperature-conditioning treatments that favor chilling tolerance, were identified in mandarins and grapefruits (Lafuente et al., 2005; Sanchez-Ballesta et al., 2006; Sapitnitskaya et al., 2006). Comparison of the results found in different citrus fruit cultivars suggested that the various temperature-conditioning treatments may induce distinct molecular mechanisms related to citrus fruit tolerance to chilling, regardless of whether they involved heat or not (Sanchez-Ballesta et al., 2003, 2004; Sapitnitskaya et al., 2006).

Information about the global mechanisms associated with cross-adaptation induced by heat to cold stress in citrus fruits is very scarce. In an early genomic approach, a suppression subtractive hybridization (SSH) cDNA library was constructed. This library was enriched in the genes induced in the flavedo of Fortune mandarins conditioned for 3 days at 37°C with HA, whose expression persisted when fruits were transferred to low temperature, and also in the genes induced by a heat+cold combination (Sanchez-Ballesta et al., 2003). About 38% of the genes in this library showed a homology with proteins of known functions. Among them, the most abundant encoded proteins involved in metabolism, plant defense responses, and transcription and signal transduction (Sanchez-Ballesta et al., 2003).

Very little information is available about the global mechanisms induced by heat or cold in the flavedo of citrus fruit that develop injury in response to chilling. Transcriptomic, proteomic, and metabolomic analyses have been performed on the pericarp and juice sacs of citrus fruit, exposed or not to heat treatments, and stored at low temperatures that did not cause CI (Perotti et al., 2011, 2015; Yun et al., 2012, 2013). As far as we are aware, only one report has compared the transcriptome profiling of the flavedo of grapefruits stored at a temperature that causes CI after being conditioned, or not, at a temperature treatment that reduces CI (Maul et al., 2008). This treatment was performed at 16°C for 7 days, so the acclimation mechanisms induced by this hardening treatment should differ, at least in part, from those related to cross-adaptation induced by heat to cold stress. Therefore, the aim of this study has been to determine global changes in gene expression that occur in Fortune mandarins, either exposed or not to a heat-conditioning treatment (HA 37°C for 3 days, curing) and stored at low temperature. Emphasis has been placed on the changes that occur in response to the heat+cold combination because the genes in this category are the best candidates to be involved in heat-induced tolerance to chilling. These genes have been named HICT, from heat-induced chilling tolerance. With this approach we will add knowledge about cross-adaptation in plants whereby exposure to one stress, like heat, provides tolerance to another, like chilling.

## Materials and methods

### Fruit and heat-conditioning and storage temperatures

Full mature fruits of the hybrid mandarin Fortune (*Citrus clementina* Hort. Ex Tanaka x *Citrus reticulata*, Blanco) were harvested in March during three citrus seasons from a commercial orchard at Castellón, Spain. By this month, Fortune mandarins had a maturity index (°Brix/acid content) higher than 12 and the fruits have reached the maximum orange peel color (h° lower than 40) (Holland et al., 1999). Fruits were selected for homogeneous size, free from defects, and immediately delivered to the laboratory. For each experiment, fruits were randomly divided in two lots per temperature assayed. The first lot was sorted into three replicates of 10 fruits each to estimate chilling damage along fruit storage. The second lot, made up of three replicates of 10 fruits per temperature and storage period, was used to evaluate changes in gene expression. Periodically, flavedo samples were collected from the total surface of fruits, frozen and homogenized in liquid nitrogen. The homogenized tissue was stored at −80°C for later analysis.

For transcriptomic analysis, Fortune mandarins were conditioned at 37°C and 90–95% RH for up to 3 days and then were stored at 2°C and 80–85% relative humidity (RH) for 60 days. Control non-conditioned fruits were stored immediately after harvest under the same storage conditions (2°C and 80–85% RH). In addition, to determine changes in the expression of genes encoding transcription factors (TFs), another group of fruits, which were not conditioned at 37°C, were stored at a non-chilling temperature (12°C) and 80–85% RH for the same storage period.

### Estimation of CI index

**Figure 1:**
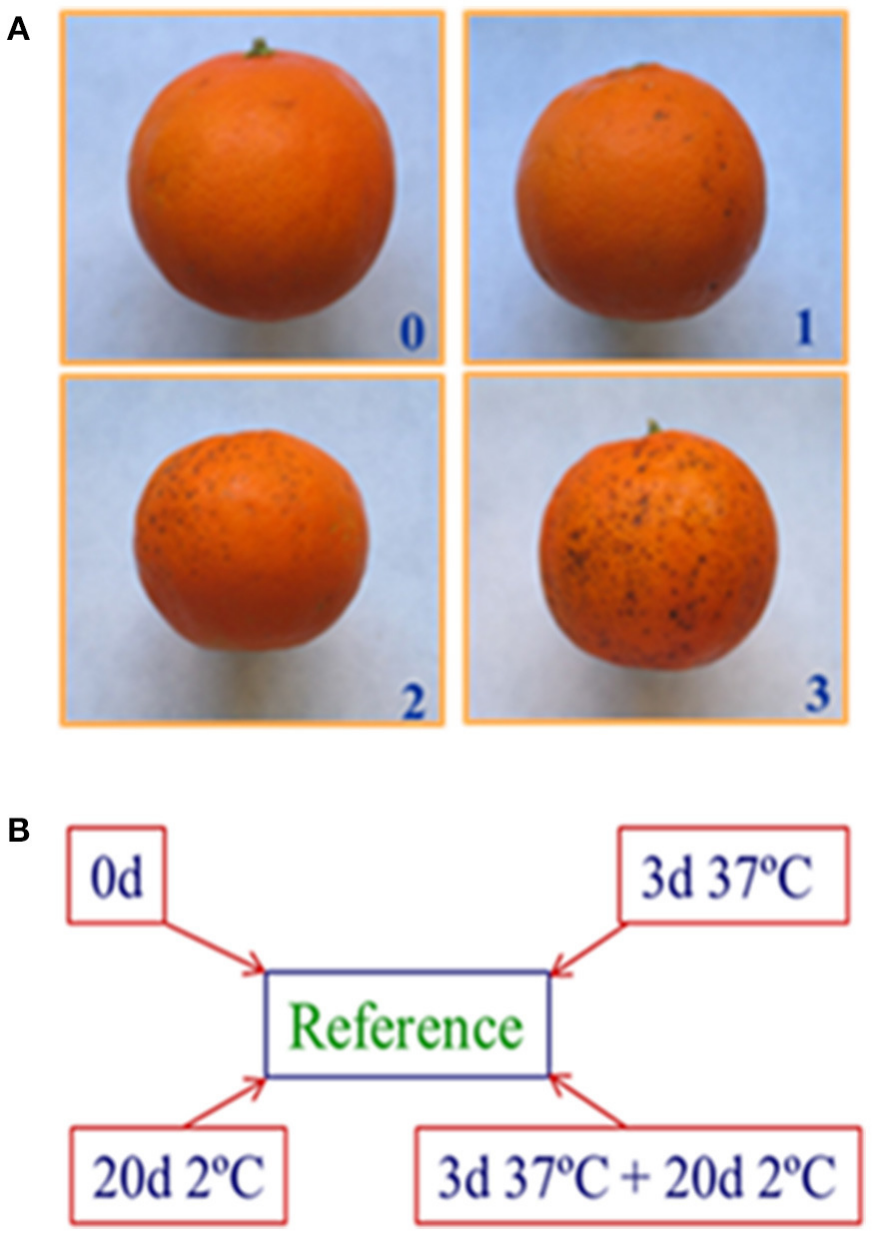
Fortune mandarin CI symptoms and their quantification and schematic diagram of the experimental design used for the analysis of the transcriptome changes. **(A)** The visual rating scale used to evaluate CI severity. **(B)** The transcriptomic analysis was performed in the flavedo of freshly harvested fruits (0 d), of the fruits exposed to heat (3 days at 37°C, curing), to cold (20 days at 2°C), and to the heat+cold combination (3 days 37°C+20 d 20°C). The cDNA samples were Cy5-labeled and co-hybridized with a Cy3-labeled cDNA reference pool from a mixture that contained equal amounts of RNA from all the assayed samples.

Brown pit like depressions in the flavedo were the main CI symptoms in Fortune mandarins. The effect of the heat treatment on the severity of cold-induced damage was evaluated on a rating scale that ranged from 0 (no injury) to 3 (severe injury) (Figure 1A), and the CI index was calculated as previously described (Lafuente et al., 1997) by summing the products of the number of fruits in each category by the value assigned to each category in the rating scale, and dividing the sum by the total number of fruits evaluated. The results are the means of three replicate samples containing 10 fruit samples ± SE.

### RNA isolation and cDNA labeling and microarray hybridization

Total RNA was extracted from frozen flavedo as previously described by Ballester et al. (2011). Possible genomic DNA contaminations were removed by treating total RNA with Ribonuclease-free DNase (Ambion/Applied Biosystems, Austin, TX, USA) following the manufacturer's instructions and RNA concentration was measured spectrophotometrically (Nanodrop, Thermo Fisher Scientific, Madrid, Spain). RNA integrity was verified by agarose gel electrophoresis and ethidium-bromide staining (Ballester et al., 2011). cDNA synthesis and purification, dye coupling, and labeled-cDNA purification were performed according to Forment et al. (2005). Three biological replicates from samples harvested during the same citrus season or during three different seasons were used for RNA isolation and the subsequent microarray hybridization.

### Microarray hybridization, data acquisition, and analysis

The analysis of the transcriptome changes that take place in the flavedo of Fortune mandarins during fruit exposure to heat (3 d 37°C), cold (20 d 2°C), and the heat+cold combination (3 d 37°C+20 d 2°C) was done to know the molecular events associated with long-term heat-induced chilling tolerance (Figure 1B). Our previous data have shown that the chilling- and the heat-induced physiological responses of citrus fruits are strongly influenced by pre-harvest factors. Therefore, a transcriptomic analysis was first performed with three biological replicate samples harvested at the same maturity stage during only one citrus season and the results were compared with those obtained by using biological replicates from fruits harvested during three different seasons at the same maturity stage.

Two microarrays were used, which were developed as part of the citrus functional genomics project (CFGP) (http://bioinfo.ibmcp.upv.es/genomics/cfgpDB/) (Forment et al., 2005), and contained about 7,000 (7 K) and 12,000 (12 K) unigenes. The samples from different citrus seasons were analyzed with the 12 K microarray (Ballester et al., 2011), which includes all the genes of the 7 K microarray. All the genes were isolated from 52 cDNA libraries of citrus that cover different tissues, a wide range of fruit varieties, developmental and fruit ripening stages, and also distinct stress conditions (Forment et al., 2005). These microarrays include the genes isolated from a cDNA library, named FlavCurFr1, from the flavedo of Fortune mandarins fruits exposed to 37°C for periods that ranged from 4 h to 3 days, and also from fruits preconditioned for 3 days at this temperature and held from 1 to 10 days at a chilling temperature (Forment et al., 2005). The cDNA samples were Cy5-labeled and co-hybridized with a Cy3-labeled cDNA reference pool from a mixture that contained equal amounts of RNA from all the assayed samples (Figure 1B). This reference sample allowed to lower the number of hybridizations to make all the possible pairwise comparisons between samples (Ballester et al., 2011). Hybridized microarrays were scanned by using a GenePix 4000A scanner (Axon Instruments, Sunnyvale, CA, USA) and only spots with a background-subtracted intensity greater than 2-fold the mean of background intensity were used for normalization and further analysis (Romero et al., 2012). A significant analysis of microarrays (SAM), included in the TM4 Microarray Software Suite, was performed to identify differentially expressed genes for all possible pairwise comparisons using a False Discovery Rate threshold *p*-value < 0.01 as previously described (Ballester et al., 2011). The identification of biological processes that were significantly under- or over-represented in a set of differentially expressed genes respect to a reference group composed of all genes in the microarray with an *Arabidopsis thaliana* homolog was carried out using the program FatiGO+ (Babelomics, http://babelomics.bioinfo.cipf.es) (Ballester et al., 2011). A Fisher two-tailed test (*p*-value < 0.05) was independently performed for gene ontology analysis of induced and repressed genes.

### Northern analysis of selected transcription factors (TFs)

Special attention was paid to transcription factors (TFs) given their relevance in modulating the expression of specific groups of genes, and also because of previous results that have highlighted the relevance of TFs in heat-induced chilling tolerance (Sanchez-Ballesta et al., 2003). A detailed study of the expression of a group of TFs (21 TFs) selected from the 50 TFs present in the FlavCurFr1 cDNA library was performed. Changes in expression levels were analyzed by Northern blot hybridization in fruits exposed for different periods at 37°C, and in the non-conditioned and conditioned (3 days at 37°C) fruits stored for up 60 days at 2°C. Moreover, changes in their expression in non-conditioned fruits stored at a non-chilling temperature (12°C) were compared with those taking place in the non-conditioned fruits stored at the chilling temperature.

Samples of denatured total RNA (10 μg) were separated on 1.2% (w/v) agarose-formaldehyde gel, transferred to a nylon Hybond–N+ membrane (Amersham Biosciences) using 20X SSC (3 M sodium chloride, 0.3 M sodium citrate, pH 7.0) as the transfer medium and cross-linked using a UV Crosslinker UVC 500 (Hoefer, Inc.). Membranes were stained with methylene blue 0.03% in 0.3 M sodium acetate pH 5.2 to measure loading variation and pre-hybridized for 2 h at 42°C in UltrahybTM Hybridization Buffer (Ambion, Inc.). Selected probes were labeled with α(^32^P)-dATP by linear amplification using a Strip-EZTM PCR Kit (Ambion, Inc.). Hybridization with selected probes was carried out overnight at 42°C (Ballester et al., 2006) and membranes washed and exposed to “Imaging Plate” (Fujifilm) film with intensifying screens. Quantification of the hybridization signals was performed with the Image Gauge Program V 4.0 (Fuji). The filters were stripped off and re-hybridized to the 26S rDNA *C. sinensis* probe as described by Ballester et al. (2006) to normalize the hybridization of the TFs genes. The ratio between the hybridization signal of each TF mRNA and that obtained using the 26S rDNA *C. sinensis* probe was calculated and transcript accumulations were normalized respect to the values found in freshly harvested fruits. A value of 100 was assigned to this sample.

## Results and discussion

### Effect of heat-conditioning on CI injury of fortune mandarins

**Figure 2:**
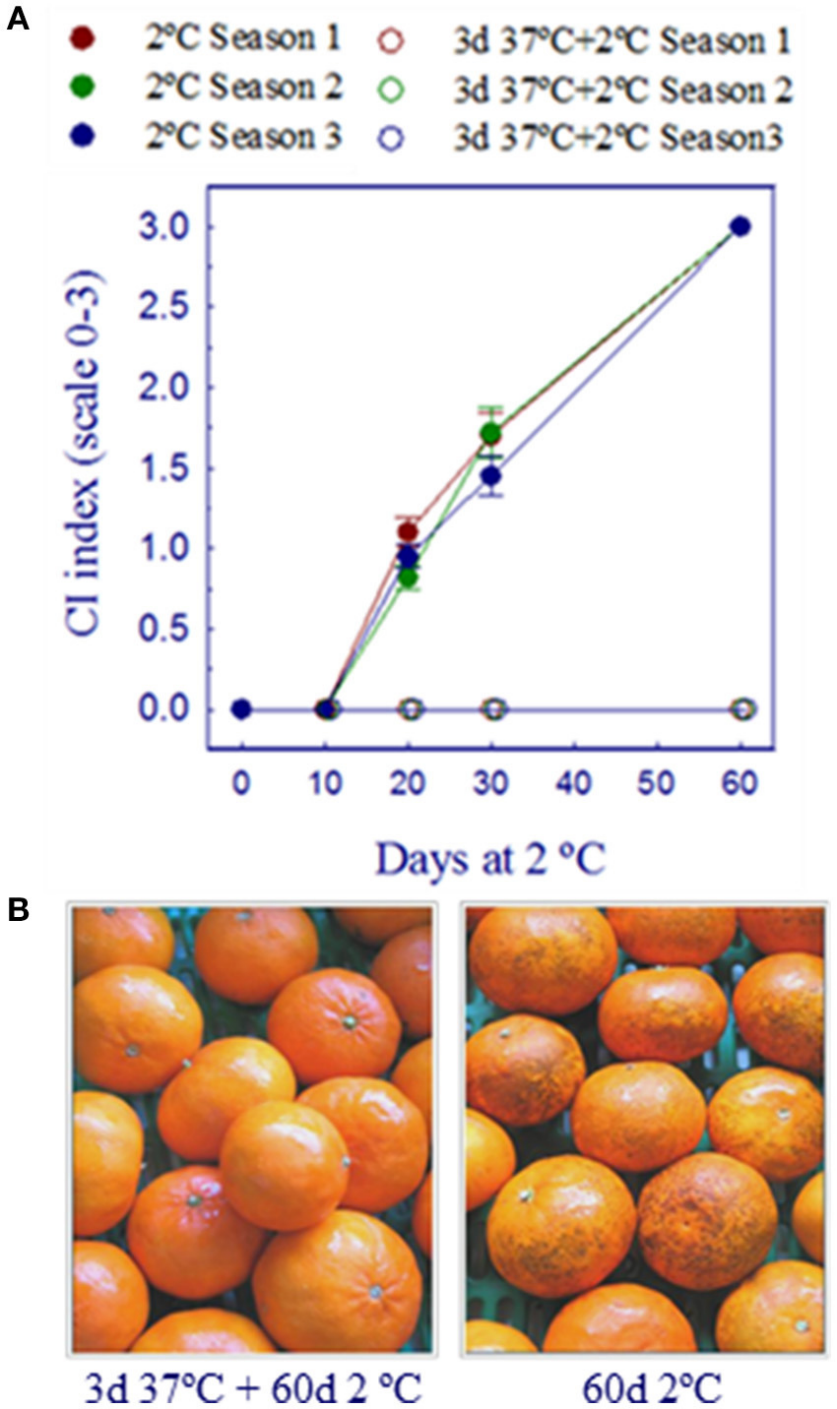
Effect of conditioning Fortune mandarins for 3 days at 37°C and 90–95% HR on chilling injury (CI) development at 2°C. **(A)** Changes in the CI index of the fruits harvested during three different citrus seasons and used as biological replicates. **(B)** The conditioning treatment overrides chilling injury (CI) development for at least 60 days.

The effect of conditioning Fortune mandarins for 3 days at 37°C was examined in fruits harvested during three different citrus seasons. As shown in Figure 2A, CI developed in the non-conditioned fruit after 10 days of cold storage (2°C). The severity of the disorder was low by day 20 (CI index of about 1 in a rating scale from 0 to 3) and by day 60 all the non-conditioned fruits showed severe damage (CI index 3). Results also showed that the susceptibility of the fruits harvested during the three citrus seasons was very similar since CI damage changed similarly during cold storage in the non-conditioned fruits. As expected, conditioning the fruits at 37°C for 3 days was always very effective reducing CI. As shown in Figures 2A,B, Fortune mandarins from the three citrus seasons did not develop CI for at least 60 days if previously cured. On the basis of these results, samples taken from the three seasons can be very useful to compare molecular responses induced by heat conditioning and cold stress in fruits showing similar chilling susceptibility but exposed to different pre-harvest conditions.

### Heat-induced transcriptomic changes in cold stored citrus fruits

The heat-conditioning treatment selected to study changes in the transcriptome was one that lasted 3 days at 37°C. The treatment was selected because of its high efficacy in reducing CI has been proven to be very reproducible throughout different citrus seasons, and it is independent of pre-harvest factors (Lafuente et al., 1997; Gonzalez-Aguilar et al., 2000). Moreover, previous physiological data have shown that shortening the duration of the conditioning period would reduce the treatment's efficacy in Fortune mandarins (Gonzalez-Aguilar et al., 1998; Lafuente et al., 2011), which suggests that the heat-induced chilling tolerance is not limited only to the transient responses induced at 37°C. The treatment allowed to extend cold storage for at least 60 days. Therefore, we focused in the molecular events associated with long-term heat-induced chilling tolerance and examined changes occurring in fruits heated for 3 days at 37°C and in conditioned and non-conditioned fruits held for 20 days at the chilling temperature (2°C) (Figure 1B). The 20 days period was selected to determine molecular responses that were induced by the heat treatment, whose expression persisted after prolonged cold storage, and that were induced by the combination of heat plus long-term storage. By this period, chilling damage started only in non-conditioned fruits. It is well-known that pre-harvest factors have a marked effect on the chilling- and the heat-induced physiological responses of citrus fruits (Lafuente et al., 1997, 2003; Gonzalez-Aguilar et al., 2000; Holland et al., 2002). Therefore, the transcriptomic analysis was first performed with three biological replicate samples harvested during one citrus season and the results were compared with those obtained by using biological replicates from three different citrus seasons. Fruits from these replicates were harvested in the same maturity stage, and showed the same susceptibility to CI (Figure 2A). In this way, our analysis is more restrictive, but the level of confidence in the molecular changes that are associated with heat-induced chilling tolerance is higher. The microarray includes genes isolated from 52 cDNA libraries of citrus, including the genes isolated from the FlavCurFr1 cDNA library. This library covers the genes induced by the heat-conditioning treatment, whose expression could persist or not when mandarins are subsequently transferred to chilling. Moreover, it covers genes that were not induced by the heat-conditioning treatment alone, but conditioning enhanced or accelerated their induction in chilled fruit (Forment et al., 2005).

**Figure 3:**
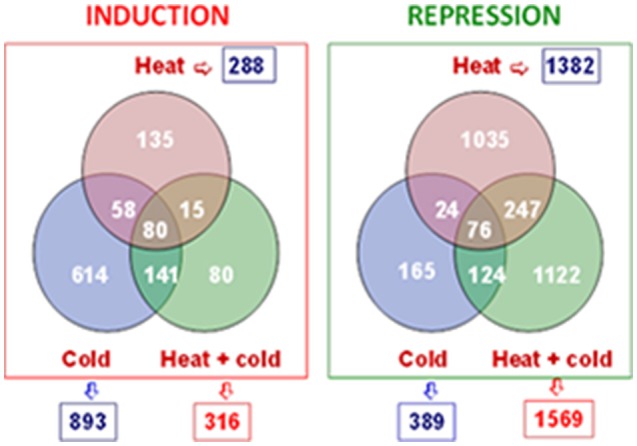
Venn diagrams showing the number of differentially expressed genes (SAM, FDR <0.01) in the flavedo of the fruits exposed to heat, cold, and heat+cold. The expression levels of the up-regulated and down-regulated genes in these samples were compared with the levels of the freshly harvested fruits. Three biological replicates from fruits harvested during different citrus seasons were used. The numbers in rectangles are the sum of all the induced and repressed genes under each particular condition.

The transcriptomic changes that occur in response to heat and cold, and the heat+cold combination, were studied. A significant analysis of microarrays (SAM) showed that 38% of the differentially expressed genes (DEGs) corresponded to the HICT category, as they responded to the heat+cold combination (Figure 3). Trascriptomic analysis also showed that repression prevailed in heat-conditioned fruits as there were many more genes repressed under this condition (1569), and also in response to heat (1382), than induced (316 and 288, respectively). In contrast, induction prevailed in the flavedo of the chilled fruits that had not been previously conditioned at 37°C (893 induced genes and repressed 389 ones).

The gene ontology analysis of DEGs in the fruits exposed to the heat treatment in non-conditioned cold-stored fruits and in fruits exposed to the heat+cold combination allowed to group DEGs in biological processes, which were over- or under-represented in response to different treatments (Table 1). The number of over- or under-represented biological processes was much lower when biological replicates from fruits harvested during three different seasons, rather than harvested the same day in one citrus season, were included in the transcriptomic analysis. This effect reflects a higher dispersion of the results as a consequence of the influence of pre-harvest factors. However, as mentioned above, the level of confidence in the processes that would be associated with heat-induced chilling tolerance would be higher. As shown in Table 1, the only common differentially expressed processes in both analysis were the lipid biosynthetic process, which was repressed by heat in cold-stored fruits compared to freshly harvested fruit, and translation, which was significantly repressed by cold stress, but only when the effect of cold was compared with the induced by heating the fruits at 37°C for 3 days (Table 1).

**Table 1:** Significant non-redundant biological processes over- or under-represented in the flavedo of Fortune mandarins exposed to heat (H), cold (C), or to the heat+cold combination (HC).

**Biological process (FatiGO+)**	**1 Citrus season**	**3 Citrus season**
**LEVEL GO 5**		
Amino acid derivative metabolic process	HC > FH	
	HC > C	
Macromolecule biosynthetic process	C&HC < FH	
Carboxylic acid metabolic process		HC < FH
**LEVEL GO 6**		
Lipid biosynthetic process	H < FH	
	**HC < FH**	**HC < FH**
	C > HC	
Translation	HC < FH	
	**C < H**	**C < H**
	C < FH	
**LEVEL GO 7**		
Isoprenoid biosynthetic process		HC < FH
**LEVEL GO 8**		
Regulation of transcription, DNA-dependent	HC > FH	
	C > FH	

*A gene ontology (GO) analysis was performed by the program FATIGO+ (FatiGO+, P < 0.05) Lower GO levels represent general biological processes, whereas higher ones denote more precise information. Symbols > and < indicate that the process was over- or under-represented under the first storage condition indicated in the comparison. The results obtained by using samples harvested from one and three citrus seasons showing the same susceptibility to CI (Figure 2A) were compared*.

### Genes involved in lipid metabolism

Many authors agree with the theory proposed by Lyons (1973), which indicates that changes in membrane fluidity induced by cold stress is the primary event related to CI in plants, and that fluidity depends on both the degree of fatty acid unsaturation and the fatty acid composition of phospholipids. This idea has been supported by the results found in different plant systems, including transgenic plants, but has also failed in numerous species (Parkin et al., 1989). Considering the relevance of lipids in membrane permeability, and our above mentioned results in heat-conditioned Fortune mandarins, special attention has been paid to changes in the expression of genes belonging to the lipid biosynthetic process.

**Figure 4:**
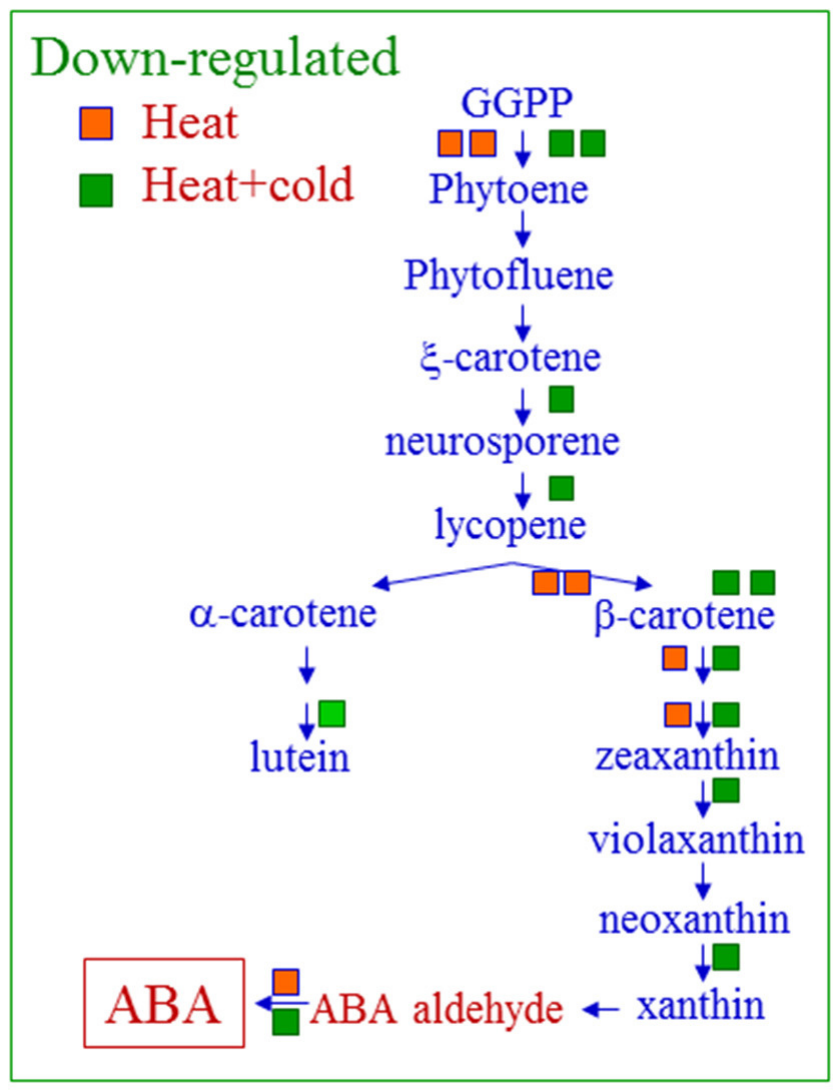
High temperature-conditioning (3 days at 37°C) down-regulates the expression of the different genes involved in the carotenoid and subsequent biosynthesis of ABA. Orange and green squares represent repressed genes in response to heat (3 days at 37°C) and the heat+cold combination (3 days at 37°C+20 days at 2°C), respectively. Major changes in gene expression levels are indicated by using two squares.

The genes related to carotenoid and epicuticular wax (CUT 1, CER) biosynthesis, and also to lipid biosynthesis or elongation, and encoding several desaturases, were found mainly among the genes included in the lipid biosynthetic biological processes repressed by the heat-conditioning treatment in cold-stressed fruit (Table S1). Carmona et al. (2006) have shown that curing treatment may affect carotenoid content and composition in sweet oranges. Likewise, Matsumoto et al. (2009) have demonstrated in Satsuma mandarins that carotenoid composition and accumulation is highly dependent on postharvest temperature, and that the synthesis of various carotenoids decreases in fruits treated at high temperature (30°C). These results agree with those found in the present work (Table S1) and summarized in Figure 4. As shown in this figure, the heat-conditioning treatment had a strong impact and down-regulated a set of genes involved in the biosynthesis of carotenoids which, in turn, are precursors of ABA (orange squares in Figure 4). This effect was enhanced after holding the previously heat-conditioned fruit at low temperature (green squares in Figure 4). These results could explain our previous data, which showed that ABA levels lowered after exposing fruits for 3 days at 37°C, and remained lower in conditioned fruits than in non-conditioned ones during storage at 2°C (Lafuente et al., 1997). Data on grapefruits have indicated that a hardening treatment of 16°C increases fruit chilling tolerance and up-regulates some genes involved in ABA biosynthesis (Maul et al., 2008). So, it is feasible to think that ABA might be involved in the efficacy of this hardening treatment in grapefruits, but not in that of the heat-conditioning treatment at 37°C in Fortune mandarins. Thus, previous physiological data, which indicate that ABA may even enhance CI in citrus, should be considered (Lafuente et al., 1997; Gosalbes et al., 2004; Alférez et al., 2005). This is in line with the findings that have indicated that ABA is not involved in low-temperature stress response in the juice sacs of cold-stored citrus fruits (Yun et al., 2012).

**Figure 5:**
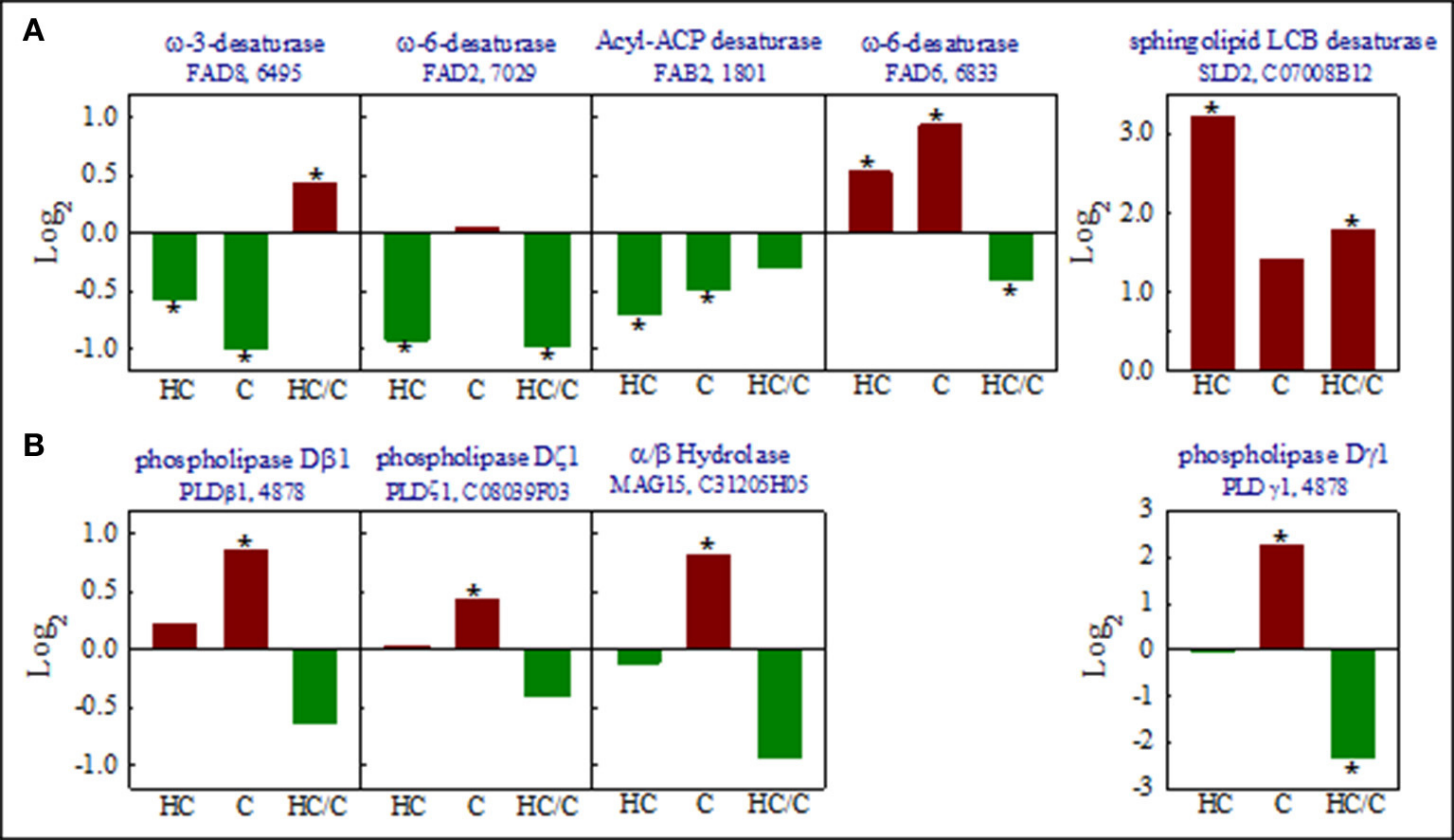
Changes in the expression levels of the genes that encode lipid desaturases **(A)** and the lipases **(B)** that occur in response to heat (H, 3 days at 37°C), cold (C, 20 days at 2°C), and the heat+cold combination (3 days at 37°C + 20 days at 2°C). Values were obtained by comparing the changes in expression levels that occurred in response to heat plus cold (HC) or cold (C) to the expression levels of the freshly harvested (FH) fruits, and by also comparing the expression levels between the heat-conditioned cold-stored fruits (HC) and the cold-stored non-conditioned fruits (C). Unigenes are indicated at the top of each graph. The asterisk indicates statistically differences in each comparison according to SAM (FDR-adjusted *p*-value < 0.01).

The study of the genes included in the lipid biosynthetic process also revealed that the expression levels of three desaturases in cold-stressed fruits were lower in the fruits previously exposed to heat treatment than in non-conditioned fruits (Figure 5A). Only the expression level of a sphingolipid desaturase (SLD2), which responds to cold stress, increased when the previous heat treatment was applied. This was a relevant effect, as heat-conditioned chilled fruit exhibited an 8-fold accumulation of this desaturase (Figure 5A). By using an Arabidopsis mutant, the *AtSLD2* gene has been found to play a key role in protecting plants against chilling (Chen et al., 2012). It is noteworthy that sphingolipids are required for the normal activity and stability of the plasma membrane, and they may act as second messengers in regulating defense responses, and are linked to redox signaling (Gechev et al., 2006). These results suggest that the degree of fatty acid unsaturation is not a limiting factor in the heat-induced chilling tolerance of Fortune mandarins. This agrees with previous physiological data, which have shown that exposing citrus fruit to high temperature, by applying the same HA (37°C for 3 days) treatment, or to an intermittent warming treatment, barely affects the degree of lipid unsaturation in cold-stored citrus fruits (Mulas et al., 1997; Schirra and Cohen, 1999). Therefore, it would seem that the degree of lipid unsaturation is not likely a critical factor in the chilling tolerance of citrus fruits. In contrast, the HA-conditioning treatment had a clear effect repressing chilling-induced increases in the expression levels of different phospholipases, especially type D phospholipases, and the expression of an aclyglicerol lipase (α/β hydrolase), which is involved in lipid degradation (Figure 5B). Although phospholipases may produce signaling molecules that play a defensive role in plants against stress cues, they also cause membrane damage if plant systems are exposed to severe or continuous stress. So, the protective effect of the heat-conditioned treatment appears to be more likely related to the repression of the genes involved in lipid degradation than to the modification of fatty acids unsaturation that affects membrane permeability. Interestingly, changes in the expression of the genes that encode desaturases are up-regulated in grapefruits exposed to the hardening treatment (7 days at 16°C), which also reduces CI, while the expression of a lipase 3 induced by chilling in this fruit barely varies by treatment (Maul et al., 2008). This further confirms that the cross-adaptive mechanism, which operates in the chilling tolerance induced by treatments that involve heat, may differ from those associated with acclimation to cold induced by a hardening process.

### Transcription factors

Special attention was also paid to TFs given their relevance in modulating the expression of specific groups of genes, and also because of results found by Sanchez-Ballesta et al. (2003) that highlighted the relevance of transcription initiation factor IIB (TFIIB) and two WRKY TFs in heat-induced chilling tolerance. Table 2 shows the results obtained from the study of changes in the expression of the TFs selected from the FlavCurFr1cDNA library (Forment et al., 2005). Changes in expression levels were analyzed by Northern blot hybridization in Fortune mandarins treated from 4 h to 3 days at 37°C, and in the conditioned (3 days at 37°C) and non-conditioned fruits kept from 1 to 60 days at 2°C, to know early and late responses to cold stress, to heat during the conditioning treatment, and to the heat+cold combination. A gene expression analysis allowed TFs to be clustered in three groups. The TFs with a higher or lower expression in heat-conditioned than in non-conditioned fruits when stored at the chilling temperature were clustered in pattern 1 and pattern 2, respectively. Therefore, the TFs in these patterns might participate in heat-induced chilling tolerance. Other genes repressed by cold stress in both the heat-conditioned and the control non-conditioned fruits, or that did not respond to either cold or heat+cold, were included in pattern 3 (Table 2).

**Table 2:** Relative expression level of the genes that encode transcription factors (TFs) isolated from the cDNA library FlavCurFr1 in the flavedo of the fruits exposed to different temperature regimes.

	**Transcript**		**37°C (H)**				**3 d 37°C + 2°C (H+C)**						**2°C (C)**						**12°C**					
**Tair10defline**	**ID**	**FH**	**4 h**	**12 h**	**1 d**	**3 d**	**1 d**	**3 d**	**10 d**	**20 d**	**30 d**	**60 d**	**1 d**	**3 d**	**10 d**	**20 d**	**30 d**	**60 d**	**1 d**	**3 d**	**10 d**	**20 d**	**30 d**	**60 d**
**Pattern 1: H+C >C**																								
TATA binding protein 2	19256625	100	73	92	126	85	75	85	**150**	**241**	125	122	**55**	64	114	92	116	102	138	**148**	135	114	116	103
WRKY DNA-binding protein 48	19260860	100	91	112	83	74	89	86	88	109	**165**	125	119	114	116	117	105	111	115	103	109	107	100	94
WRKY DNA-binding protein 28	19279147	100	94	113	71	65	96	132	**205**	**260**	**215**	111	82	66	93	**55**	72	73	122	123	**175**	**144**	**179**	**152**
WRKY transcription factor 40	19252004	100	124	93	92	77	106	124	102	**44**	**40**	68	65	81	**43**	70	65	107	**41**	**46**	**59**	**43**	**55**	**167**
Transcription factor IIA, alpha/beta subunit	19282074	100	69	90	79	69	92	84	95	**160**	**199**	**259**	**147**	126	138	**168**	**184**	**177**	112	**143**	136	120	126	105
**Pattern 2: H+C ≤ C**																								
Transcriptional corepressor SEUSS	19272965	100	95	79	77	67	87	86	86	94	101	120	135	**147**	**162**	**148**	**148**	**141**	106	109	108	117	98	94
Auxin response factor 19	19262959	100	81	95	69	71	93	66	71	86	93	104	95	70	118	**147**	**171**	**186**	101	**146**	132	139	107	**151**
Zinc finger protein, LSD1-type	19286170	100	131	106	97	101	117	128	**167**	**263**	**284**	**259**	**141**	**147**	**218**	**220**	**291**	**281**	116	126	114	110	94	93
Telomere repeat-binding protein 1	19283888	100	**47**	**40**	**35**	**28**	**38**	**58**	136	**280**	**144**	**143**	125	**261**	**440**	**245**	**290**	**177**	126	106	99	87	88	63
RING/U-box superfamily protein	19262758	100	65	63	**30**	**31**	**44**	**58**	108	**231**	**295**	**230**	125	**191**	**327**	**224**	**319**	**246**	**146**	128	102	80	87	63
RING-H2 finger protein RHC1a	19287094	100	98	110	**153**	94	89	103	80	66	63	107	123	126	139	132	**170**	**183**	**141**	**140**	106	109	114	105
Transcription factor bHLH113	19276300	100	116	**145**	**145**	133	124	105	68	**44**	**49**	**56**	129	111	66	73	74	91	**58**	**52**	65	66	68	64
**Pattern 3: H+C = C**																								
DREB subfamily A-4 of ERF/AP2 transcription factor family (ERF026)	19275624	100	103	94	79	92	**48**	**32**	**36**	**39**	**44**	**37**	**42**	**41**	**30**	**50**	64	98	**50**	**52**	83	**52**	67	**365**
Heat Stress Transcription Factor (Hsf) family	19276116	100	**59**	64	**38**	**37**	**44**	**41**	**47**	**44**	**37**	**35**	67	65	**57**	**52**	**56**	**60**	60	**52**	**48**	**52**	**43**	75
Knotted1-like homeobox gene 3	19261948	100	76	85	64	62	68	**59**	63	**56**	**52**	**47**	99	87	63	**53**	**54**	72	96	107	119	108	93	109
Calcium-binding transcription factor involved in salt stress signaling	19276886	100	108	94	68	60	**55**	**50**	**51**	**45**	**57**	81	74	73	62	**56**	75	98	77	**59**	**51**	63	61	109
Transcription factor, MADS-box	19279286	100	82	71	**50**	**39**	**42**	**42**	**35**	**25**	**21**	**27**	97	84	67	**54**	61	63	119	128	119	132	130	119
Component of the FAcilitates Chromatin Transcription (FACT) complex	19285547	100	83	66	78	**45**	**33**	**37**	**37**	**35**	**38**	**45**	81	74	66	**54**	65	77	108	75	66	71	**60**	109
B-box containing transcriptional regulator	19280828	100	69	85	70	70	71	62	70	67	117	85	79	70	63	86	**58**	65	85	100	103	105	102	82
RING/U-box superfamily protein	19259847	100	81	89	66	60	70	66	72	89	92	82	90	86	80	76	86	92	108	101	96	109	87	128
Multiprotein bridging factor 1	19268326	100	120	**144**	**172**	96	88	91	87	73	66	72	88	97	99	82	89	74	**196**	**178**	**163**	**150**	139	**147**
**Chilling injur index (scale 0–3)**		0.0	0.0	0.0	0.0	0.0	0.0	0.0	0.0	0.0	0.0	0.0	0.0	0.0	0.0	0.6	1.6	2.5	0.0	0.0	0.0	0.0	0.0	0.0

*Transcript accumulations were normalized respect to values found in the freshly harvested fruits (FH), and a value of 100 was assigned to this sample. Changes in expression were analyzed in fruits exposed to heat (H, 37°C) from 4 h till 3 days; in the non-conditioned (C) and in heat-conditioned fruits (3 days at 37°C) (H+C) maintained from 1 to 60 days at 2°C, and in the fruits maintained at a non-chilling temperature (12°C) for the same storage periods. Changes in CI index are shown at the end of the Table. Inductions are marked in red (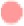 > 140–200; 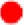 > 201) and repression in green (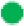 < 60). Genes were grouped in three patterns of expression. Pattern 1 and 2 group the genes whose expression was higher (>) or lower (<), respectively, in cold-stored fruits that were previously heat-conditioned (H+C) at 37°C for 3 days. Pattern 3 groups the genes repressed by cold stress in both the heat-conditioned and the control non-conditioned fruits, or that did not respond either to cold or heat plus cold*.

Within the expression pattern 1, which included the HICT genes, three WRKY TFs were identified. Two presented a similar expression pattern, with maximum mRNA accumulation in the heated fruit maintained at 2°C for 20–30 days, when CI became evident in the non-conditioned fruits, but not in the heat-pretreated fruits (Table 2). Their expression pattern was especially interesting as their relative accumulation increased by the heat+cold combination despite transcripts levels not rising in response to cold or heat. WRKY TFs are one of the largest families of transcriptional regulators found only in plants. Nowadays, diverse biological functions have been described for these TFs, including cold and heat tolerance (Bakshi and Oelmüller, 2014). Although very little is known about the participation of the WRKY family in the tolerance of plants to chilling (Bakshi and Oelmüller, 2014), these results reinforce our previous idea and highlight the relevance of WRKY TFs in the cross-protection induced by heat against chilling in citrus fruits (Sanchez-Ballesta et al., 2003). In line with this, it should be pointed out that the WRKY TFs shown in Table 2 differed from those previously reported as HICT TFs (Sanchez-Ballesta et al., 2003), and that all the selected TFs that encoded WRKYs were included in the same expression pattern. Moreover, the three WRKYs within pattern 1 were found only in the library FlavCurFr among the 52 cDNA libraries constructed in the CFGP, which covers a wide range of tissues, varieties, developmental, and fruit ripening stages and stress conditions (Forment et al., 2005). Heat-conditioning also favored the cold induction of two components of the RNA polymerase II transcription machinery in the eukaryotes that belong to the TFIID complex (initiation complex TFIID), which is required for transcription initiation and activated transcription in plant cells (Pan et al., 2000), a TATA-binding protein and a TFIIA factor. As shown in Table 2, the expression of these genes was higher in the conditioned fruits after 20 and 60 days of cold storage, respectively. Moreover, the transcriptome analysis revealed that a gene that encodes a TFIIE factor was expressed only in the fruits exposed to the heat+cold combination. These results are in line with the idea that the TFs that are relevant for the transcriptional and translational apparatus of plant cells are also important in heat-induced chilling tolerance. The transcriptomic analysis also showed that the HA-conditioning treatment favored the up-regulation of genes involved in ethylene signaling, of ethylene-responsive transcription factors (EIN4, EIN 3, EREBT), and of TFs that belong to the zinc finger, NAC/NAM and Myb/Myc families in fruits stored at low temperature (Table S1). However, only relevant differences were found in expression levels of a gene of the NAC family protein. The participation of the above mentioned HICT genes in inducing tolerance against chilling appears to be specifically associated with heat treatment since genes from these families are not related to acquiring the chilling tolerance induced at 16°C in grapefruits (Maul et al., 2008). Therefore, protection strategies induced by both temperature-conditioning treatments to cope with citrus fruit tolerance to chilling are likely to differ. The cultivar effect should also be considered. In fact, in non-conditioned grapefruits, cold stress favors the repression of some WRKYs (Maul et al., 2008), with minor changes occurring in response to cold stress in the Fortune mandarins that were not previously conditioned (Table 2).

Other TFs were induced in response to low temperature in non-conditioned fruits and the mRNA accumulation of these genes was totally, or to some extent, repressed if fruits were preconditioned at 37°C (pattern 2, Table 2). The expression of these genes generally increased with CI development for up to 30 days. This behavior may indicate that their up-regulation is not necessary in heat-conditioned fruit to cope with cell damage since the HA treatment avoids development of CI. However, we cannot rule out that heat-induced damage reduction may be associated with the repression of these genes in cold-stored citrus fruits. They encode proteins of different families, including zing finger proteins, MYB, a co-regulator transcriptional SEUSS, and an auxine response factor (ARF), whose expression only increases in non-conditioned fruits. The role of this hormone in the chilling tolerance of citrus fruits is unknown, but auxines may play a protecting role against oxidative stress (Kovtun et al., 2000), which has been associated with CI in Fortune mandarin (Sala and Lafuente, 1999; Sanchez-Ballesta et al., 2003) and other citrus cultivars (Sala et al., 2005; Rivera et al., 2007; Ghasemnezhad et al., 2008; Maul et al., 2008; Lado et al., 2016). The transcriptomic analysis also showed slight differences in the expression of two TFs from the YABBY and two from the HSTF families, which showed a higher expression in cold-stored fruits if they had not been previously conditioned at 37°C.

The TFs within pattern 3 (Table 2) do not appear relevant for the cold acclimation of Fortune mandarins to chilling, although some genes from these TFs families may play important roles in the defense of plants and fruits against various stresses (Sevillano et al., 2009). Most of these genes are down-regulated by cold stress in both heat-conditioned and non-conditioned fruits, despite the fact that some including CBF1, MADS-box, MYC, or HSFs, belong to the gene families related to cold-stress acclimation in grapefruits or other chilling-sensitive crops (Maul et al., 2008; Sevillano et al., 2009; Peng et al., 2015).

These results thus reinforce the idea that the molecular mechanisms that operate in the defense of citrus fruits against chilling differ with the conditioning treatment type. Moreover, they agree with the idea raised by Wang et al. (2001) from their research in apple fruit cells that suggests that the protective effect of heat treatments may be related to an increased transcription capacity in cold-stressed cells (Wang et al., 2001). The global results from changes in expression of TFs also reflect that long-term heat-induced chilling tolerance in citrus fruit is an active process that requires new transcription factors in cold-stored fruits from the WRKY family. Many genes from this family can be induced by cold and heat stress in Arabidopsis (Bakshi and Oelmüller, 2014) and, interestingly, the WRKY HICT identified in citrus fruits were up-regulated only by the heat+cold combination. Therefore, these genes could be good candidates to be involved in heat-induced tolerance against chilling in citrus fruits, and further research should be conducted to decipher their regulatory role in this process.

### Major changes in gene expression in miscellaneous categories

**Figure 6:**
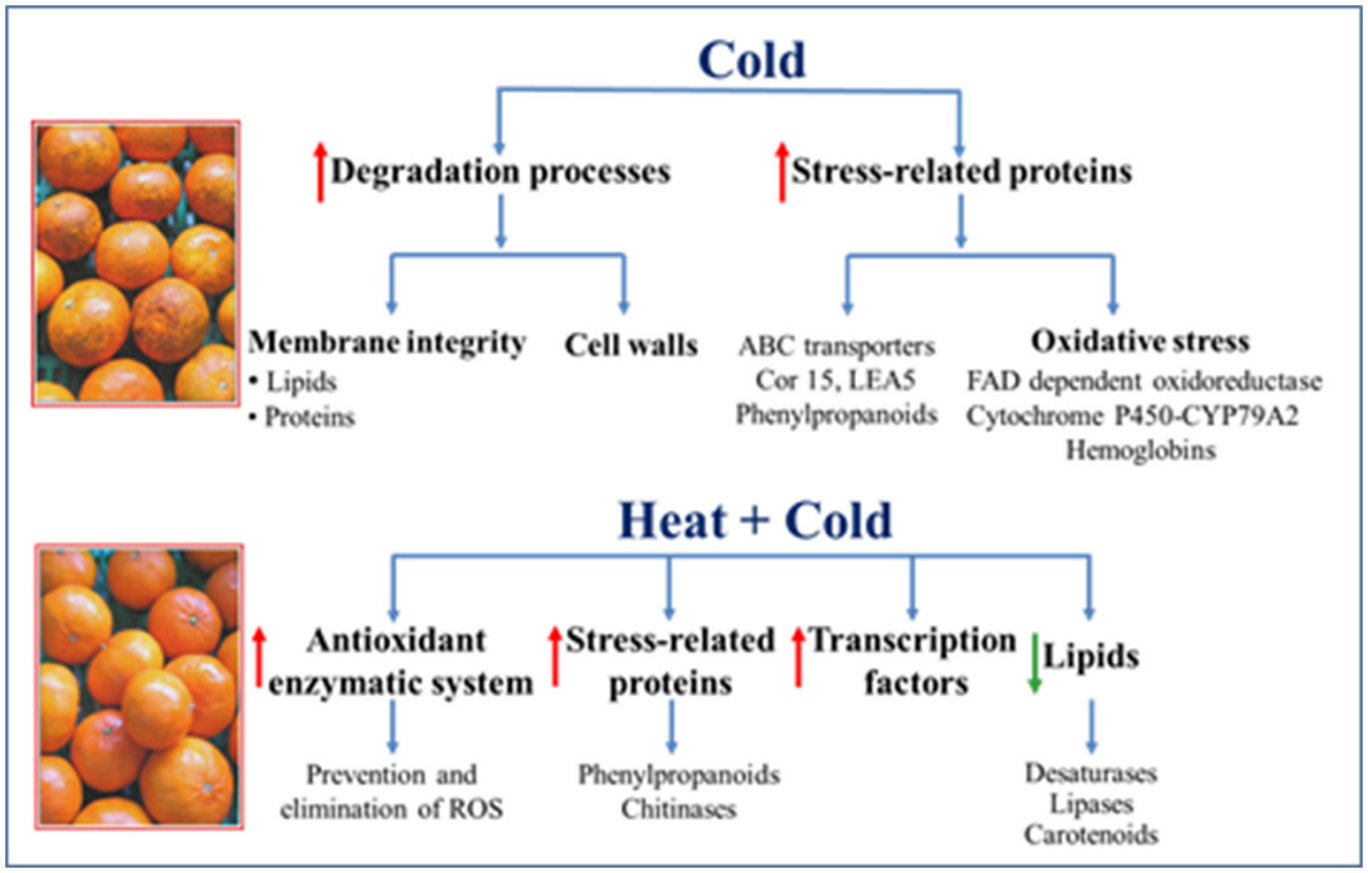
Summary of the cold-activated (↑) or repressed (↓) responses in the non-conditioned (Cold) and heat-conditioned (Heat+cold) Fortune mandarins.

The transcriptomic analysis also provided very valuable information about other genes that may be related to the tolerance or susceptibility of citrus fruits to chilling. Changes in the expression levels of these genes were relevant, although the number of genes within a specific category was not high enough to reveal the over-representation of a specific biological process. Such information is shown in Table S1 and summarized in Figure 6. From such data, it can be pointed out that cold stress in non-conditioned chilled citrus fruit favors membrane integrity loss as it promotes degradation processes that affect both lipids and proteins, as we identified rises in lipases (Figure 5), and also in some proteases, from which the most relevant changes took place in cysteine proteases. Furthermore, cold stress induced marked increases in the expression levels of genes that encode cell wall-degrading enzymes, like a β-polygalacturonase encoding genes and a β-xylosidase, whose expression was at least 4-fold higher in cold-stored non-conditioned fruits. Interestingly, it has been recently shown that cell wall-derived oligomers reduce CI in citrus fruits (Vera-Guzman et al., 2017).

Chilling also had a strong effect by up-regulating a set of genes related to stress responses, many of which are involved in oxidative stress. It induced relevant changes in the expression levels of not only several ABC transporters, whose expression increased by up to 9-fold, but also of cold responsive (COR) genes and dehydrins, which could help fruit to cope with chilling stress. Among them, the expression of *cor15* genes was higher in non-conditioned fruits, which reinforces the idea exposed by Sanchez-Ballesta et al. (2004) that these proteins are not related to heat-induced chilling tolerance in citrus fruits. Likewise, the expression of different genes that encode LEA proteins increased in response to cold stress, and most of them were repressed by heat in cold stored fruits. Although these proteins have been linked mainly to water stress, they are induced in response to diverse stresses, including cold and oxidative stress (Mowla et al., 2006). Chilling also up-regulated the expression of different genes belonging to the phenylpropanoid metabolism, which is in agreement with findings in cold-stressed mango indicating that chilling stress activates phenylpropanoid pathway (Sivankalyani et al., 2016). Moreover, it had an important impact increasing expression level of oxidative-stress related genes in non-conditioned fruits. Among them, we found a high representation of genes encoding FAD-dependent oxidoreductases and of cytochrome P450 (monooxygenase-1electron) related-proteins, which were down-regulated by the heat-conditioning treatment. Among the FAD-dependent oxidoreductases, the marked rises in nectarin 5 (NEC5) and CPRD2 were noteworthy, whose expression increased by more than 8-fold in response to cold stress. These genes are involved in the accumulation of ROS and membrane damage. Likewise, a very high increase (9–16-fold) was observed in various cytochrome P450-encoding CYP79A2 proteins. These proteins lead to the synthesis of glycosylated compounds that contain sulfur and display antioxidant activity. These genes were among the most induced in non-conditioned cold-stored mandarins and its expression was down-regulated if the fruits were previously treated for 3 days at 37°C. Genes encoding hemoglobin were also among the most cold-induced genes in non-conditioned mandarins. A 25-fold increase in expression levels of two hemoglobins was found. While these proteins are widespread in the plant kingdom, their function is still not well-understood. However, it has been suggested that they may provide an alternative type of respiration to mitochondrial electron transport under limiting oxygen concentrations and also modulate nitric oxide levels in stressed plants (Dordas, 2009).

On the other hand, from the results obtained with the transcriptomic analysis, it can be pointed out that heat-induced chilling tolerance in citrus fruit seems to be an active process requiring, besides new transcription factors, the activation of stress-related proteins and the repression of genes favoring tissue damage (Figure 6). Many of the genes that encode stress-related proteins were involved in the prevention or elimination of ROS and in secondary metabolism. Within oxidative stress, major increases (10-fold) in the expression levels of a gene that encodes a ferritin, which could sequester ferrous ions, were observed. Therefore, the HA treatment could reduce oxidative stress by preventing the Fenton effect and the subsequent formation of hydroxyl radicals (Gechev et al., 2006). This highly toxic specie may cause severe oxidative damage to lipids, but also to DNA and proteins. The analysis has also shown that high-temperature conditioning might protect citrus fruit against CI by scavenging ROS through enzymes like SOD, glutathione transferase (GST), glutaredoxin, tioredoxin, as well as different metalloproteins (Table S1). These results add further knowledge about the enzymes that protect cold-stored citrus fruit from oxidative stress, and demonstrate that such protection in citrus fruits is not limited only to the traditional SOD, catalase, peroxidase, and Halliwell-Asada cycle antioxidant enzymatic system (Sala and Lafuente, 2000; Sanchez-Ballesta et al., 2003; Rivera et al., 2007; Ghasemnezhad et al., 2008; Siboza et al., 2014; Lado et al., 2016). Likewise, the heat-conditioning treatment repressed lipases and hydrolases in cold-stressed fruits and could, therefore, reduce ROS formation associated with membrane damage. The effect of the heat-conditioning treatment on inducing chitinases was also remarkable since the expression of different genes of this multigene family increased in response to both heat and the heat+cold combination. The induction of these genes in response to heat, and persistence during fruit cold storage, support previous data obtained by an SSH approach. With this approach, an acidic chitinase class II was identified as a HICT gene (Sanchez-Ballesta et al., 2003); and a high correlation has been found between the induction of this gene and the chilling tolerance induced by different temperature-conditioning treatments that display diverse efficacy against chilling (Lluch, 2006). That chitinase shows high homology with one of those identified herein by means of a transcriptomic analysis. The role of chitinases in the defense of citrus fruits against chilling remains unknown. By using an Arabidopsis mutant, it has been shown that they may play a role against different stresses like heat, dehydration, and salt stress (Kwon et al., 2007). This effect has been related to the participation of chitinases in the formation of cell walls and in adhesion between cell membranes and the cell wall. Moreover, Gao and Showalter (1999) have reported that the alteration of these glycoproteins leads to cell death in Arabidopsis cells. After considering these results, and that membranes are the first cell component affected by cold stress, the study of the role of chitinases in the chilling tolerance of citrus fruits and other horticultural crops deserves further attention. Within the secondary metabolism, the effect of the heat-conditioning treatment increasing the expression levels of diverse genes of the phenylpropanoids metabolism in cold-stored fruits was remarkable. The genes that encode cinnamate 4-hydroxylase and isoflavone reductase proteins, and four oxygen methyl transferases (OMTs), showed higher expressions in heat-conditioned fruits than in the non-conditioned ones stored at low temperatures. Conversely, the expression of a gene that encodes PAL, which catalyzes the first phenylpropanoid biosynthesis step, remained unaltered in the heat-conditioned fruits maintained under chilling conditions, but increased in response to cold stress in the non-conditioned fruits (Table S1). Therefore, the beneficial effect of curing treatment is more likely to be related to the metabolic shifts of phenylpropanoids by leading to the synthesis of both flavonoids and methylated phenypropanoid compounds than to increasing phenolics content. OMTs perform diverse functions in plants. In the present work, it should be mentioned that they are the principal enzymes in the complex network of reactions that occur as part of lignin biosynthesis, but OMTs may also lead to the synthesis of coumarins, which display antioxidant activity (Lee and Jang, 2015). Therefore, heat-conditioning could have an effect on cell wall fortification, but also on increasing levels of the natural compounds in the flavedo with antioxidant activity. Accordingly, Yun et al. (2013) found that a HWD treatment performed at 52°C for 2 min up-regulated stress response proteins that belong to the secondary metabolism in the citrus pericarp. Although these authors did not examine the maintenance of these responses after transferring fruits to cold stress, it is interesting to note that the short heat treatment was able to increase lignin content in the pericarp.

Chilling increases ethylene production in citrus fruits (Martinez-Tellez and Lafuente, 1997). Previous reports have indicated that this phytohormone plays a protective role against chilling in Shamouti oranges and Fortune mandarins, since applying inhibitors of ethylene action, such as 1-methylcyclopropene (1-MCP), and synthesis increase chilling-induced damage (Porat et al., 1999; Lafuente et al., 2001). However, low 1-MCP levels may reduce the incidence of the disorder in other citrus cultivars (Salvador et al., 2006). Other results have shown that ethylene production transiently increases at 37°C during the 3-day conditioning treatment (Holland et al., 2012), which markedly reduces CI. However, the conditioning treatment prevented the rise in ethylene that occurs in non-conditioned fruits in response to chilling, which was much higher than that induced at 37°C (Holland et al., 2012). This result might suggest that during the 3 days that the conditioning treatment at 37°C lasts, some defense mechanism against chilling is initiated and, therefore, the cold-induced increase in ethylene is not necessary to cope with stress or to contain lesion propagation in tissues that present CI. Global results thus suggested that ethylene may participate in the defense of citrus fruits against chilling, but does not play a critical role in reducing CI. In the present work, we found very marked changes in the genes that encode FAD-dependent oxidoreductases, in abundant GSTs, ABC transporters, and also in different transcription factors, which were only induced in non-conditioned fruits in response to cold stress (Table S1). These genes are regulated in citrus fruits by ethylene (Establés-Ortiz et al., 2016). However, the comparison of the changes in the transcriptome of the flavedo of chilling-exposed fruits with those previously identified in our laboratory when studying the effect of exogenous ethylene (Establés-Ortiz et al., 2016) have shown that the percentage of genes regulated by both chilling and ethylene did not exceed 15% (Establés-Ortiz, 2008). Therefore, this is in line with our previous idea that most protective mechanisms of citrus fruits against chilling do not depend on ethylene.

## Conclusion

Heat conditioning is a very effective reproducible method to reduce CI in citrus fruits. Transcriptomic data has revealed that although some responses to heat and cold temperatures in citrus fruits are common, specific responses toward each condition prevail. Moreover, data highlight that the heat+cold combination induces a high proportion of specific responses. Hence, these responses should be the best candidates to be involved in the heat-induced chilling tolerance in citrus fruits. Inhibition of cold-induced responses may play an important role in the events that control heat-induced chilling tolerance. In fact, gene repression prevails in heat-conditioned chilled fruits, while induction prevails in non-conditioned cold-stored fruits. The study also highlights the importance of pre-harvest environmental conditions on heat and/or cold-induced responses. Moreover, comparison of results from this study with previously reported data in citrus fruits shows the importance of varietal differences, and that the events that control the chilling tolerance induced by distinct temperature-conditioning treatments, may differ. These results envisage that the CI problem is not a simple one and that the primary CI event in citrus fruits remains unknown. Three major factors seem to correlate with the chilling tolerance induced by preconditioning citrus fruit at high temperature: (1) repression of genes involved in membrane degradation; (2) activation of the responses that aim to prevent oxidative damage; (3) activation of the HICT TFs of the WRKY family involved in transcription initiation.

## Author contributions

ML reviewed the literature and wrote the paper and designed the experiments leading to data shown. BE carried out such experiments and participated in data analysis. LG contributed to drafting the manuscript as well as to the design of the experiments and to data analysis. All authors read and approved the final manuscript.

### Conflict of interest statement

The authors declare that the research was conducted in the absence of any commercial or financial relationships that could be construed as a potential conflict of interest.

## Supplementary material

Table S1 Fortune mandarin transcript expression profile in heat-conditioned (3 days at 37°C) and non-conditioned fruits stored for 20 days at 2°C. Sheets 7 and 12 k show the results of the hybridizations with the 7 and 12 k microarrays, respectively. The sheet “readme” explains data shown in both sheets.

## Abbreviations

ABA: abscisic acid
CI: chilling injury
CFGP: citrus functional genomic project
DEGs: differentially expressed genes
GST: glutathione transferase
HA: hot humid air
HICT: heat-induced chilling tolerance
HSP: heat-shock protein
HWD: hot water dip
SAM: significant analysis of microarrays
1-MCP: 1-methylcyclopropene
OMTs: oxygen methyl transferases
ROS: reactive oxygen species
PAL: phenylalanine ammonia-lyase
SOD: superoxide dismutase
SSH: suppression subtractive hybridization
TFs: transcription factors.
